# Lack of pollinators selects for increased selfing, restricted gene flow and resource allocation in the rare Mediterranean sage *Salvia brachyodon*

**DOI:** 10.1038/s41598-024-55344-7

**Published:** 2024-02-29

**Authors:** Boštjan Surina, Manica Balant, Peter Glasnović, Andrej Gogala, Živa Fišer, Zlatko Satovic, Zlatko Liber, Ivan Radosavljević, Regine Classen-Bockhoff

**Affiliations:** 1 Natural History Museum Rijeka, Lorenzov Prolaz 1, 51000 Rijeka, Croatia; 2 Faculty of Mathematics, Natural Sciences and Information Technologies, University of Primorska, Glagoljaška 8, 6000 Koper, Slovenia; 3 Institut Botànic de Barcelona (IBB, CSIC–Ajuntament de Barcelona), Passeig del Migdia S.N., Parc de Montjuïc, 08038 Barcelona, Spain; 4 Slovenian Museum of Natural History, Prešernova cesta 20, P.O. Box 290, 1001 Ljubljana, Slovenia; 5 Department of Plant Biodiversity, University of Zagreb, Faculty of Agriculture, Svetošimunska Cesta 25, 10000 Zagreb, Croatia; 6 Centre of Excellence for Biodiversity and Molecular Plant Breeding, Svetošimunska Cesta 25, 10000 Zagreb, Croatia; 7 Division of Botany, Department of Biology, Faculty of Science, University of Zagreb, Marulićev Trg 9A, 10000 Zagreb, Croatia; 8 Institute of Organismic and Molecular Evolution, Johannes Gutenberg-University Mainz, 55099 Mainz, Germany

**Keywords:** Pollination biology, *Salvia*, Population genetics, Pollen limitation, Nectar robbery, Habitat fragmentation, Population genetics, Plant ecology, Plant evolution, Plant reproduction, Community ecology, Conservation biology, Evolutionary ecology, Phylogenetics, Population genetics

## Abstract

Range contraction and habitat fragmentation can cause biodiversity loss by creating conditions that directly or indirectly affect the survival of plant populations. Fragmented habitats can alter pollinator guilds and impact their behavior, which may result in pollen/pollinator limitation and selection for increased selfing as a mechanism for reproductive assurance. We used *Salvia brachyodon*, a narrowly distributed and endangered sage from eastern Adriatic, to test the consequences of range contraction and habitat fragmentation. Molecular data indicate a severe and relatively recent species range reduction. While one population is reproductively almost completely isolated, moderate gene flow has been detected between the remaining two populations. The high pollen-to-ovule ratio and the results of controlled hand pollination indicate that *S. brachyodon* has a mixed mating system. Quantitative and qualitative differences in the community and behaviour of flower visitors resulted in limited pollination services in one population where no effective pollinator other than pollen and nectar robbers were observed. In this population, self-pollination predominated over cross-pollination. Various environmental factors, in which plant-pollinator interactions play a pivotal role, have likely created selection pressures that have led to genetic and phenotypic differentiation and different resource allocation strategies among populations.

## Introduction

Habitat fragmentation is widely recognised as one of the greatest global causes of biodiversity loss, triggering profound changes in terrestrial ecosystems^1,2^. It leads to isolation and edge effects, creating conditions that affect the survival of many species directly or by disrupting species interactions, such as those between plants and animals ^3–7^. Fragmented habitats can shift pollinator guilds^8^, alter pollinator foraging and affect pollinator behaviour^9–12^. Breakdown of plant–pollinator interactions leads not only to lower pollinator density and visitation frequency^13–15^, but also to lower pollination efficiency^16–18^. In addition, isolated, small plant populations with low specimen density can affect pollinator availability, reduce pollination success and thus interrupt pollen mediated gene flow, which may even trigger species extinctions^19–21^. Pollinator availability and pollination failure are usually associated with pollen/pollinator limitation, defined as reduced fruit and seed production due to scarce pollen supply^22^. However, pollen limitation can also select for increased autogamy as a mechanism of reproductive assurance, when it increases average seed production beyond what is produced in the absence of autogamy^23^. Under these circumstances, autogamy, at least in some years, may help explain the maintenance of mixed mating systems in a plant population^24–26^.

**Figure 1:**
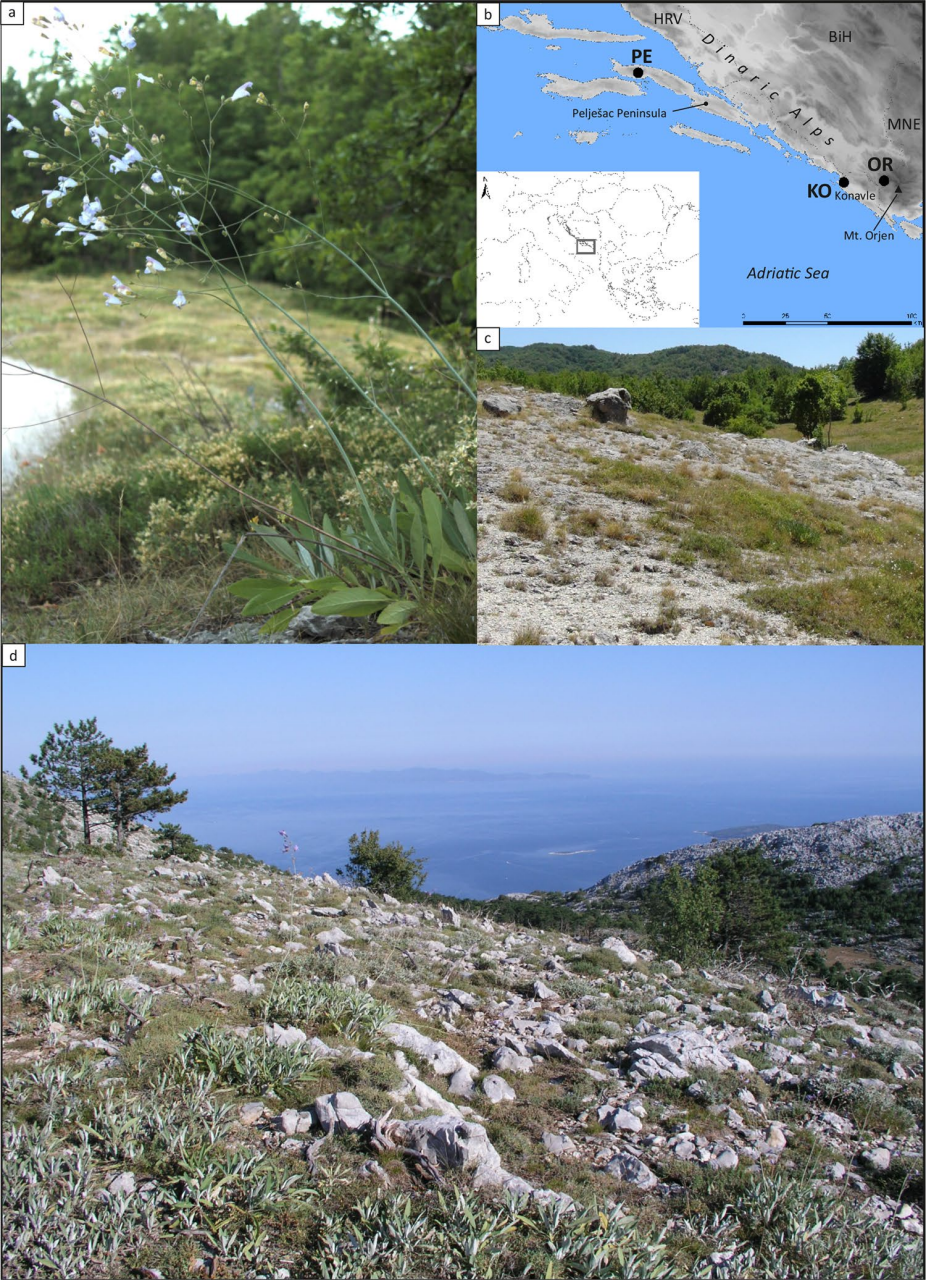
Habitus, distribution and habitat preferences of *Salvia brachyodon*. (**a**) Habitus of the plant. (**b**) Distribution area. (**c**) Dry (sub)Mediterranean grassland with *S. brachyodon* on Mt. Orjen. (**d**) Population of *S. brachyodon* (lower left-handed corner) on Pelješac peninsula with remnants of the *Pinus nigra* stands destroyed by fire. BiH–Bosnia and Herzegovina, HRV–Croatia, MNE–Montenegro. OR–population on Mt. Orjen (BiH), KO–population Konavle (HRV), PE–population on Pelješac peninsula (HRV).

**Figure 2:**
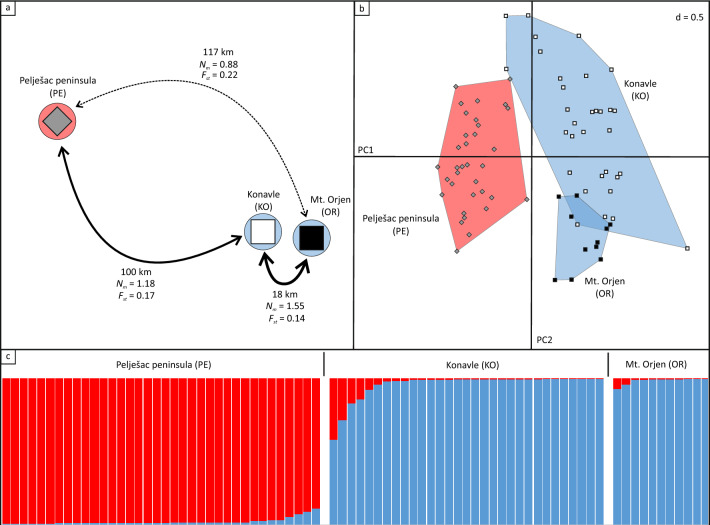
Population genetics of *Salvia brachyodon.* (**a**) Approximate geographic positions of studied populations of *Salvia brachyodon*: Pelješac peninsula–PE, Konavle–KO, Mt. Orjen–OR, with indicated distance (km) and gene flow (*N*_*m*_) between populations. (**b**) Principal component analysis (PCA) using the first and the second axis of specimens of all extant populations based on microsatellite data. (**c**) Genetic structure and assignment of individuals into classes as assessed by the computer program Structure. Each individual specimen is represented by a single vertical line; each colour represents a cluster, and the length of the coloured segments indicates the individual’s estimated proportions of membership in those clusters. Population’s symbols (enlarged) in (**a**) follow those in (**b**), while arrow thickness in (**a**) indicate intensity of gene flow. Colour codes in (**b**) are congruent with the results of genetic structuring (**c**).

It is widely accepted that generalists are less affected by habitat fragmentation than specialists and that habitat fragmentation often leads to the extirpation of specialists followed by an influx of generalist species^27–29^. However, the specialization of plant-pollinator interactions is asymmetric and the plant specialization cannot be considered in isolation from the degree of specialization of the mutualist partners^30^. Nevertheless, disruption of pollination and seed dispersal mutualisms involving specialists and/or keystone species is particularly problematic because of the potential cascading effect on the rest of the community^10^. One species that is characteristic of specific sub-Mediterranean rocky grassland communities in the eastern Adriatic is *Salvia brachyodon* Vandas (Fig. 1a). It is a narrowly distributed and endangered Mediterranean sage (Fig. 2b) that occupies dry (sub)Mediterranean grasslands (Fig. 2c and d) with highly fragmented populations^31^. We selected all currently known populations of *S. brachyodon* to test the hypothesis that the species suffers from habitat contraction and fragmentation. Specifically, we asked: (a) what is the extent of gene flow among populations? (b) Are there differences in floral biology, pollination ecology, and mating systems between populations? (c) Is there evidence of pollen/pollinator limitation? (d) What is the difference in degree of ecological specialization between populations? (e) Does self-pollination, as opposed to cross-pollination, lead to trade-offs in flower size and seed weight? To assess genetic structuring and gene flow among populations, we used plastom sequences and DNA fingerprinting data in all extant populations, while two of the three extant populations were used to study the mating system by in situ controlled hand pollination. We examined floral morphology and biology, seed weight, and visitor/pollinator assemblages to address the problem of resource allocation triggered by the shift from outcrossing to autogamous selfing and the extent of phenotypic differentiation and ecological specialization. Since *S. brachyodon* is an endangered species, our study additionally aimed to identify the source population for its possible translocation activities at potentially suitable sites.

## Results

### Population genetic structuring and gene flow

From 265 analysed samples, 78 multi-locus genotypes (MLGs) were recognized. In population OR, only 11 MLGs were identified, while 36 and 31 MLGs were detected in populations PE and KO, respectively. Following the number of detected MLGs, slightly higher levels of observed and expected heterozygosity and allelic richness were detected in populations PE and KO if compared to population OR which was seemingly characterized by depleted levels of genetic variability (Table 1). Nevertheless, no departures from Hardy–Weinberg equilibrium were detected in any of the studied populations. The fixation index (*F*_st_) values were significant for all pairs of populations. The strongest differentiation was detected between populations PE and OR (*F*_st_ = 0.22), the weakest between populations KO and OR (0.14), while the fixation index value of 0.17 was detected for the populations PE and KO (Fig. 2a). Accordingly, the highest values of gene flow (*N*_*m*_) were obtained between populations OR and KO (1.55), and the lowest between populations OR and PE (0.88), while value between populations KO and OR was 1.18. The results of *F*_st_ statistics and gene flow followed relative geographic distances between pairs of populations. Results of both PCA (Fig. 2b) and Bayesian assignment test (Fig. 2c) support the presence of two gene pools (genetic clusters). The highest *ΔK* was obtained at K = 2 (2195.7), while the *ΔK* at K = 3 was 203.1. The larger genetic cluster is comprised of MLGs from populations KO and OR, while genotypes from population PE form the other one. The lengths of the *rpl*32–*trn*L and *rps*16-*trn*K sequences were 796 and 685 bp, respectively. No nucleotide substitutions and indel events were detected.

**Table 1:** Sampling localities, number of individuals and population genetic parameter estimates based on microsatellite markers of *Salvia brachyodon.*

Population	Lat/Long (VGS84)	Elevation (m)	*N*_smpl_	*N*_pg_	*H*_o_	*H*_e_	*F*_is_	Gene bank accession numbers	Vouchers
Pelješac peninsula (HRV)—PE	42° 59′ 45.9″/17° 09′ 15.5″	910	119	36	0.71	0.70	− 0.01	OQ067506; OQ067515	NHMR3176
Konavle (HRV)—KO	42° 35′ 55.2″/18° 14′ 32.2″	490	70	31	0.61	0.62	0.02	OQ067501; OQ067510	NHMR3174
Mt. Orjen (BiH)—OR	42° 34′ 20.9″/18° 26′ 57.8″	820	76	11	0.58	0.57	− 0.02	OQ067504; OQ067513	NHMR3175

BiH—Bosnia and Herzegovina, HRV—Croatia, *N*_smpl_—number of sampled individuals, *N*_pg_—number of samples for population genetic analysis per population, *H*_o_—detected heterozygosity, *H*_e_–expected heterozygosity, *F*_is_—inbreeding coefficient. Gene bank accession numbers of *rps*16*-trn*K; *rpl*32*-trn*L.

### Flower life-span and sexual functioning

**Figure 3:**
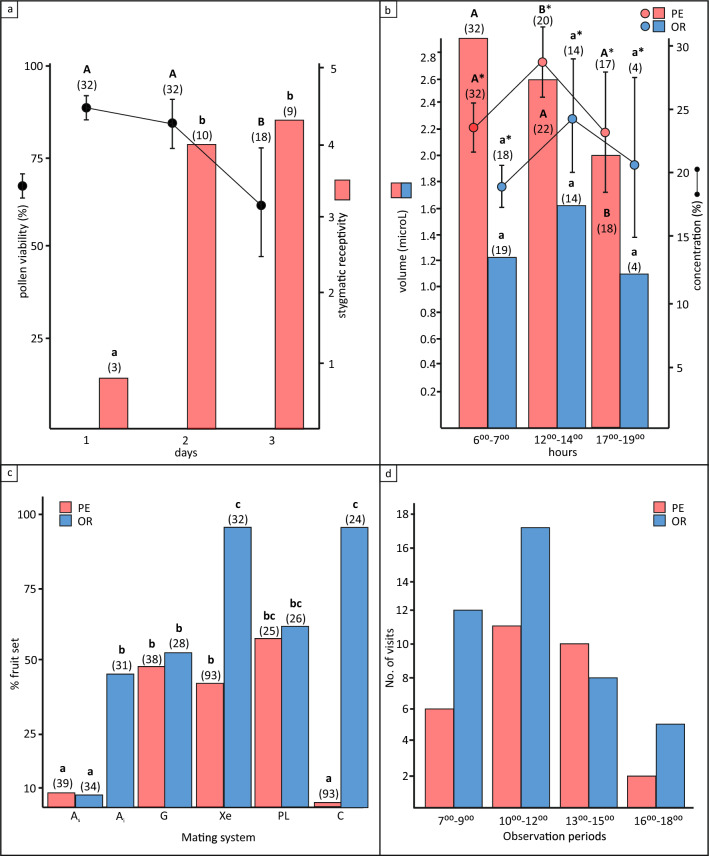
Flower biology, mating system and insect visitation of flowers of *Salvia brachyodon.* (**a**) Sexual functioning of flowers of *Salvia brachyodon* from population Pelješac peninsula–PE*.* (**b**) Nectar volume and concentration produced by flowers during the day in population PE and population Mt. Orjen–OR. (**c**) Controlled hand pollination experiments conducted to study the mating system and to assess pollen limitation for populations PE and OR. Pollination treatments: A_s_–spontaneous selfing, A_i_–induced selfing, G–geitonogamy, Xe–xenogamy, PL–pollen limitation, C–control. (**d**) Mean insect visitation frequency of flowers during the 2 days in four observation periods in populations PE and OR. Different letters indicate statistically significant differences in male and female function according to flower age (**a**) and nectar volume and concentration (**b**; *p* < 0.05). Numbers in parentheses indicate the number of flowers manipulated (**a**–**c**).

Mean flower life-span of *S. brachyodon* was one day (1.15 ± 1.27, 97; mean ± SE, *n*) in unbagged control flowers, and up to three days in the bagged test flowers (Fig. 3a), and did not differ between populations PE (*n* = 45) and OR (*n* = 52; χ^2^ = 0.81, *p* = 0.36).

**Figure 4:**
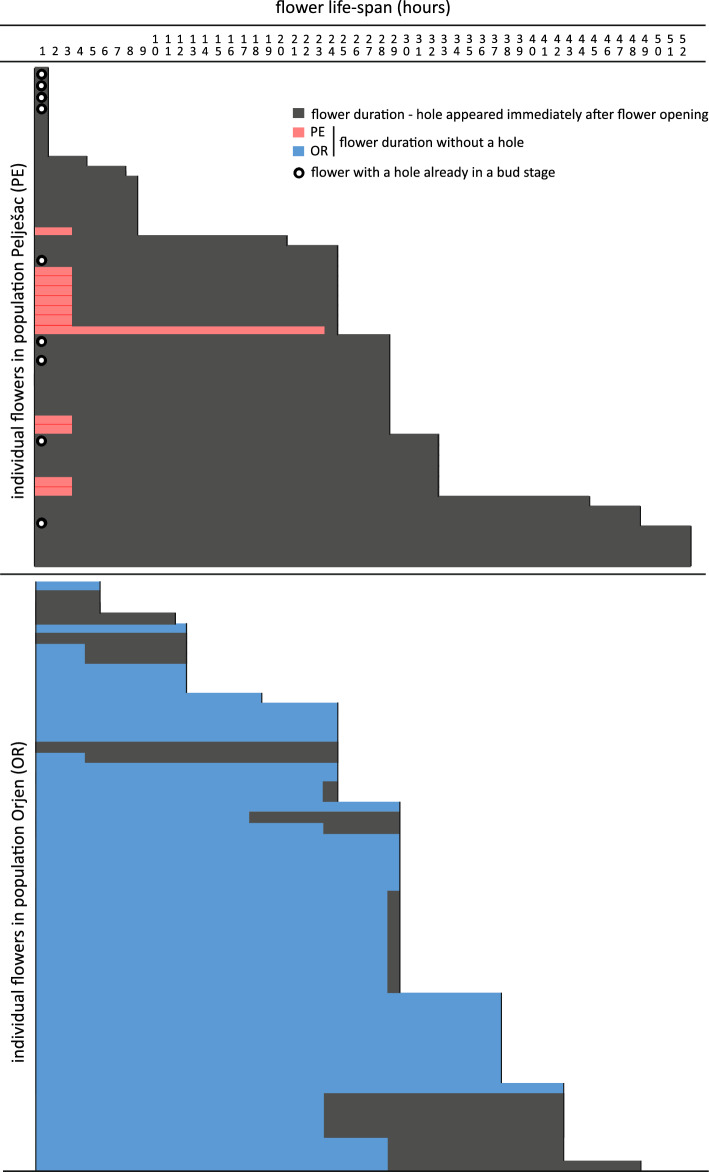
Flower life-span (hours) of *Salvia brachyodon* according to the first occurrence of a hole drilled by nectar robbers. Populations Pelješac (PE, *n* = 45, red bars) and Mt. Orjen (OR, *n* = 52, blue bars).

At population PE, 41 flowers (91%) had a hole in a corolla tube, 9 already in the bud stage, 4 of which subsequently wilted immediately. The number of flowers damaged by nectar robbers was significantly lower in population OR (58%, χ^2^ = 13.29, df. 1, *p* < 0.001), where no holes were detected in the bud stage (Fig. 4). The anthers dehisced immediately after flower opening, and pollen was generally available in the anthers until the second day, when it was almost completely depleted by floral visitors in most observed flowers. The highest pollen germination was observed on the first day when flowers opened (mean = 87%), and decreased steadily (83%) until the second day of anthesis and significantly (68%; *p* < 0.05) on the third day. Low stigma receptivity was observed on the first day (12%), but increased significantly (*p* < 0.05) with the onset of stigma bifurcation on the second (80%) and third day (85%). The data reveal weak protandry with an overlap of both functions on days two and three of flower life-span, coinciding with high pollen germinability and stigma receptivity.

### Floral rewards

Daily nectar production per flower averaged 5.8 ± 0.48 µl (min = 0.8, max = 14.4) and 2.6 ± 0.34 µl (min = 0.15, max = 6.4) in populations PE and OR, respectively, and the difference between populations was significant (χ^2^ = 19.21, *p* < 0.001). In population PE, a slight decrease in nectar production was observed in the middle of the day and a significant one (χ^2^ = 5.435, *p* < 0.05) between 17^00^ and 19^00^ in the afternoon (Fig. 3b), while in population OR no significant differences in nectar reward were observed during the day. Sugar concentrations significantly differed between populations (χ^2^ = 15.2, *p* < 0.001), and averaged 24.9% ± 0.74 in population PE and 21.1% ± 1.56 in population OR. Nectar standing crop varied within and between populations, too. In population PE, nectar volume was measured in 48.4% of sampled flowers at 8^00^ and in 38.7% at 14^00^ of sampled flowers. Nectar volume was significantly higher at 8^00^ (1.26 ± 0.18 µl, 15; mean ± SE, *n*) than at 14^00^ (0.54 ± 0.19 µl, 12; χ^2^ = 9.152, *p* < 0.01). No nectar was detected in un-bagged flowers at population OR.

### Mating system, pollen limitation and pollen-to-ovule ratio

Pollination treatments significantly affected fruit set in both studied populations (χ^2^ = 180.47, df. = 10, *p* < 0.0001; Fig. 3c, Supplementary Table S1). Spontaneous autogamy (A_s_) at populations PE (8%) and OR (6%) resulted in significantly fewer fruits than any other treatment except the control treatment (C) at population PE (4%). Compared to A_s_, the induced autogamous treatment (A_i_; performed only in population OR) produced more fruits (42%, *p* < 0.05). Fruit set in geitonogamous treatments (G) were not significantly different from treatments A_i_ and pollen limitation (PL: 64% and 69% for populations PE and OR, respectively) in both populations, but was significantly higher than treatment C in population PE and lower than the xenogamous treatment (Xe) in population OR (88%). The highest fruit set (88%) was obtained with treatments Xe and C in population OR. With the exception of treatment A_i_ (OR), plants in population OR had higher or significantly higher fruit set compared to plants in population PE, regardless of the treatment applied. Inbreeding coefficients (*δ*_*i*_) were -0.07 in populations PE and 0.39 in population OR. The number of pollen grains produced per flower in population PE was 64,789 ± 10,902 (N = 10), of which 65% were in the upper theca and 35% in the lower theca of the anthers. The mean value of P/O ratio was 16,197 ± 2,725 (log_10_16,197 = 4.2).

### Flower visitors

In total, we observed 71 individuals of 24 different insect species on the flowers of *Salvia brachyodon* of populations PE and OR (Table 2). Most visitors belonged to solitary (18 species) or social bees (5 species), and only one butterfly (Sphingidae) and one wasp (Vespidae, Eumeninae) species were observed. According to their behaviour, pollen thieves dominated, represented in most cases by small solitary bees (14/24 species), followed by both nectar and pollen thieves and nectar thieves (4 and 3 species, respectively). Primary nectar robbers, which gain access to nectar through a hole in the corolla tube, were represented by three short-tongued bees—*Bombus pascuorum*, *B. humilis* and *Xylocopa violacea* (Video S1), while individuals of *B. terrestris* (Video S2) behaved as nectar thieves and robbers. The only flower visitors that effectively triggered the staminal lever mechanism and successfully deposited conspecific pollen on the receptive surface of the stigma in flowers of *S. brachyodon* were those of *Bombus argillaceus* (Video S3). In population PE, an individual of *Episyrphus balteatus*, a marmalade hoverfly (Syrphidae), was recorded with its parasite foraging for pollen between the observation periods (and thus the record not included in the analysis; Video S4). In both populations, the frequency of flower visits was highest between 10^00^ and 12^00^ and higher in the morning (7^00^–9^00^) than in the evening (16^00^–18^00^; Fig. 3d). While the number of different insect taxa visiting the flowers of *S. brachyodon* was nearly identical at each locality (14 and 13 in populations PE and OR, respectively), the insect fauna differed significantly in composition (χ^2^ = 55.313, df. 23, *p* < 0.001), flower visitation frequency, Shannon diversity (*p* = 0.02), dominance (*p* = 0.0008) and evenness (*p* = 0.01). Although *S. brachyodon* flowers were visited by insects in the population OR almost twice as often as in the population PE, most flowers in the population OR were visited by individuals of *B. argillaceus*, as evidenced by the significantly higher dominance and Shannon diversity index and the significantly lower evenness index for the population OR. Between the two populations, the flowers of *S. brachyodon* shared visitors (nectar robbers, thieves, and pollen thieves) from only three taxa (12.5% of total pollinator assemblage), and the only pollinator, *B. argillaceus*, was observed only on flowers of population OR.

**Table 2:** Floral visitors and their behaviour observed on flowers of *Salvia brachyodon.*

Floral visitors	Flower visitor behaviour	Pelješac peninsula (PE)	Mt. Orjen (OR) BiH
Observation date		HRV 20. & 21.7.2017	13.7. & 14.7.2017
Hymenoptera			
*Apis mellifera* Linnaeus 1758	PT/NT		4♀
*Bombus argillaceus* (Scopoli 1763)	EP		31♀, 14♂
*Bombus humilis* Illiger 1806	NR		9♀
*Bombus pascuorum* (Scopoli 1763)	NR		1♀
*Bombus terrestris* (Linnaeus 1758)	NT/NR	6♀	5♀
*Ceratina chalybea* Chevrier 1872	PT	2♀	2♀
*Ceratina cucurbitina* (Rossi 1792)	PT	16♀	
*Ceratina cyanea* (Kirby 1802)	PT	2♀	
*Halictus fulvipes* (Klug 1817)	PT/NT	6♀	
*Halictus kessleri* Bramson 1879	PT		3♀
*Halictus patellatus* Morawitz 1873	PT	2♀	
*Halictus scabiosae* (Rossi 1790)	PT	2♀	
*Lasioglossum convexiusculum* (Schenck 1853)	PT/NT	1♀, 2♂	
*Lasioglossum morio* (Fabricius 1793)	PT		2♀
*Lasioglossum politum* (Schenck 1853)	PT		4♀
*Lasioglossum puncticolle* (Morawitz 1872)	PT		3♀
*Megachile pilicrus* Morawitz 1878	PT	6♀	
*Megachile pilidens* Alfken 1924	NT	2♂	
*Rhodanthidium septemdentatum* (Latreille 1809)	PT	3♀	
*Scolia hirta* (Schrank, 1781)	PT	1♀	
*Stelis nasuta* (Latreille 1809)	PT		2♀
*Xylocopa violacea* (Linnaeus 1758)	NR	2♀, 1♂	4♀
Vespidae, Eumeninae sp.	NT		3♀
Lepidoptera			
Sphingidae sp.	NT	1	
Diptera			
*Episyrphus balteatus* (De Geer, 1776)*	PT	2♂	
Number of floral visitors (individuals)		14 (54)	13 (87)
Dominance* (*p* = 0.0008)		0.13	0.29
Eveness* (*p* = 0.01)		0.72	0.48
Shannon diversity index (*p* = 0.0182)		2.38	1.82

*recorded outside observation periods; ns—non significant; PT—pollen thief, NT—nectar thief, NR–nectar robber, EP—effective pollinator; BiH—Bosnia and Herzegovina, HRV—Croatia.

### Floral morphology and seed weight

**Figure 5:**
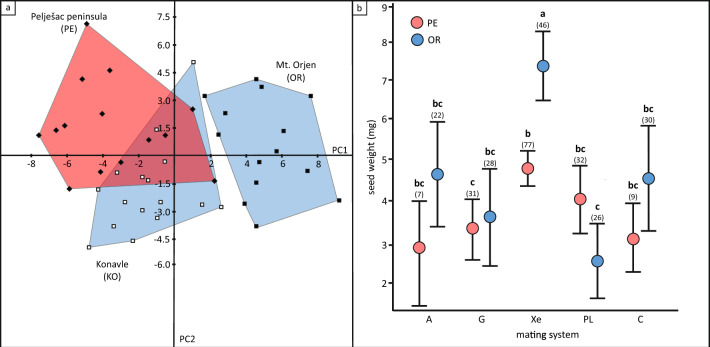
Flower morphometrics and seed weight of *Salvia brachyodon.* (**a**) Principal component analysis of flower morphometric data from all extant populations of *S. brachyodon* (Pelješac–PE, Konavle–KO, and Mt. Orjen–OR); eigenvalues and % of explained variance: 1–18.1, 34.1%, 2–9.9, 18.6%, 3–7.4, 14%, 4–6.8, 12.8%. (**b**) Seed weight of *Salvia brachyodon* according to hand pollination treatments; whisker type: SE, whisker length: 95% interval. A—autogamy, G—geitonogamy, Xe—xenogamy, PL—pollen limitation, C—control. Numbers in parentheses indicate the number of weighted seeds per treatment. Different letters indicate statistically significant differences between the treatments (*p* < 0.05). Colour codes are congruent with the results of genetic structuring in Fig. 1c.

Flowers between populations differed significantly in 11 of the 16 measured characters (Table 3, Fig. 5a). In general, the largest character values were observed in the OR population, while the smallest were observed in the PE population. The flowers of population OR differed significantly from the flowers of populations KO and PE in several characteristics. They were longer (character 1), had larger sepals (2 & 3) and corolla tubes (4, 5 & 7), greater spacing between the inner hairs of the corolla tube and the nectaries (8), and the labium and lower thecae (11). Also, the distances between the lower and upper thecae (15) were the largest in the OR population. On the other hand, the length (2) and width (3) of the sepals, the length of the corolla tube (4), and the distance between the hairs of the inner corolla tube and the nectaries (8) were the smallest in the population PE. Most of the flower characteristics measured in the KO population were between the values obtained from the flowers in the OR and PE populations. The least variable characters were corolla length (1, coefficient of variation = 4.4–14.5%), upper theca length (14, 8.6–11.6%), corolla tube length and width (4, 8.6–18.2% and 5, 8.8–15.9%, respectively), and calyx length (2, 10.2–12.1%), while the most variable characters were the distance between the upper theca and the stigma (16, 38.2–58.4%), the distance between the stigma tip and the labium (12, 19.7–43.3%), and the length and width of the labium (9, 19.3–36.6% and 10, 14.9–25.1%, respectively). Mean values of seed weight among the different populations ranged from 2.7 to 7.4 mg (Me = 3.9 mg; min. indiv. value 0.1 mg, max. 9.7 mg; Supplementary Table S2). Seed mass differed significantly between pollination treatments at both sites (χ^2^ = 65.52, *p* < 0.001; Fig. 5b). In populations PE and OR, selfing (treatments A and G) as opposed to outcrossing (Xe) resulted in lighter seeds, although significantly higher values (*p* < 0.05) were obtained only in treatment Xe in population OR. Seeds from treatment G in population PE were significantly lighter (*p* < 0.05) than those from treatments Xe in populations PE and OR.

**Table 3:** Flower characteristics (in mm) and results of the Kruskal–Wallis test (χ^2^) for the populations of *Salvia brachyodon.*

Flower character	χ^2^	populations		
		Mt. Orjen (OR)	Konavle (KO)	Pelješac peninsula (PE)
1. Flower length	20.26***	(33.8) 37.8^a^ (39.6) 4.4%	(31.0) 34^b^ (36.8) 4.7%	(28.5) 32.8^b^ (46.7) 14.5%
2. Calyx length	23.74***	(8.8) 10.5^a^ (13.7) 10.5%	(6.8) 9.2^b^ (10.5) 10.2%	(6.6) 8.1^c^ (10.0) 12.1%
3. Calyx width	25.64***	(5.3) 6.1^a^ (8.7) 14.0%	(4.5) 5.2^b^ (6.5) 10.8%	(3.7) 4.4^c^ (5.2) 11.3%
4. Corolla tube length	7.013*	(12.6) 16.2^a^ (18.7) 8.6%	(14) 16.1^a^ (18.8) 8.7%	(13) 15.1^b^ (23.3) 18.2%
5. Corolla tube width (middle)	15.57***	(9.0) 10.3^a^ (12.1) 8.8%	(6.8) 8.6^b^ (10.5) 12.2%	(6.6) 8.5^b^ (10.8) 15.9%
6. Corolla tube (distal part) width	4.258^ns^	(4.3) 6.3^a^ (7.7) 18.3%	(4.5) 5.6^b^ (8.1) 16.7%	(4.2) 5.4^b^ (8.2) 19.3%
7. Corolla tube (distal part) height	18.89***	(9.8) 11.7^a^ (13.8) 9.0%	(7.0) 8.9^b^ (12.2) 16.4%	(7.8) 9.4^b^ (11.8) 14.1%
8. Inner corolla tube hairs-nectaries distance	18.68***	(4.0) 5.4^a^ (7.9) 17.9%	(5.5) 6.6^a^ (9.0) 15.3%	(2.8) 4.7^b^ (6.6) 21.5%
9. Labium length	1.142^ns^	(5.0) 8.4^ns^ (11.6) 22.1%	(0.3) 7.3^ns^ (10.5) 36.6%	(6.4) 8.6^ ns^ (13.0) 19.3%
10. Labium width	4.132^ns^	(8.7) 14.1^ns^ (18.8) 22.2%	(2.4) 11.9^ns^ (15.1) 25.1%	(11.1) 13.6^ns^ (18.7) 14.9%
11. Labium-lower theca distance	27.65***	(2.7) 3.8^a^ (5.4) 21.4%	(1.0) 1.9^b^ (2.5) 21.5%	(2.2) 2.9^c^ (4.1) 19.0%
12. Stigma tip-labium distance	1.808^ns^	(5.0) 7.2^ns^ (9.5) 19.7%	(2.5) 6.1^ns^ (9.9) 34.1%	(1.9) 6.9^ns^ (10.7) 43.3%
13. Style exertion	17.61***	(4.8) 8.3^a^ (10.7) 23.9	(2.5) 5.1^b^ (8.4) 32.6%	(6.6) 8.0^a^ (9.6) 11.6%
14. Upper theca length	19.22***	(4.3) 5.0^a^ (5.8) 8.9%	(3.6) 4.2^b^ (5.6) 11.6%	(3.8) 4.1^b^ (5.1) 8.6%
15. Upper-lower theca distance	14.17**	(4.6) 5.9^a^ (7.2) 12.3%	(2.9) 4.7^b^ (6.3) 19.1%	(4.3) 5.0^b^ (6.0) 9.2%
16. Stigma-upper theca distance	3.298^ns^	(0.0) 3.7^ns^ (6.1) 58.4%	(1.0) 3.5^ns^ (6.9) 55.2%	(2.4) 4.8^ns^ (8.2) 38.2%

Different letters reveal statistically significant differences (sequential Bonferroni significance; *p < 0.05, **p < 0.01, ***p < 0.001, ^ns^*p* > 0.05; ns—non significant) in flower characters using Mann–Whitney pairwise post hoc test. BiH—Bosnia and Herzegovina, HRV—Croatia (min, mean and max; coefficient of variation in %; 14 flowers per population, 1 flower per genet).

## Discussion

The absence of polymorphisms in analysed plastome regions indicate a relatively recent and severe bottleneck event. Such a result was somewhat unexpected because in closely related *S. officinalis,* cpDNA loci were characterized by high polymorphism levels^32^. The ecologically similar and co-occurring *Edraianthus tenuifolius*^Table 1 in^ ^31^ experienced range contraction during the Last Glacial Maximum, but subsequently expanded its range north-westward along the climatically buffered eastern Adriatic coast^33,34^. However, the range of *S. brachyodon*, which was possibly more widespread in the past, has remained restricted and without evidence of recent expansion, contributing to the fate of Mediterranean flora with extremely high rates of narrow endemism^35,36^. Indeed, historical literature data report the occurrence of the plant approximately 80 km north of population PE^37^. However, recent confirmations of this occurrence are lacking. It remains intriguing that, despite the presence of suitable habitats along the eastern Adriatic coast, albeit fragmented, the species has remained restricted to only three extant populations.

The results of the microsatellite data analyses (Fig. 2, Table 1) indicate moderate gene flow between populations OR and KO, suggesting recent distribution range and habitat fragmentation and/or the existence of some undetected bridging populations enabling gene exchange between them. At the same time, population PE remains substantially more reproductively isolated. Such a finding was additionally supported by both Bayesian assignment test and PCA results (Fig. 2b and c) that indicate the existence of two well-defined genetic clusters, one comprising populations KO and OR, and another only population PE. The continued encroachment of deciduous woody species into calcareous/dolomitic rocky grasslands and habitat loss present a threat to all *S. brachyodon* populations, but is particularly evident in population KO^31^, where the number of plants in sample plots for genetic analyses declined from 70 + to only a few in just five years (personal observation). An excess of homozygotes is usually a consequence of increased levels of breeding among closely related individuals. However, our data suggest that fragmented and spatially isolated population KO did not suffer from reduced genetic diversity or an excess of homozygotes. It appears that even moderate pollen-mediated gene flow can contribute to long-term population viability, as demonstrated in *Ziziphus lotus* (Rhamnaceae), a sclerophyllous shrub that occurs in semiarid habitats throughout the Mediterranean region^38^. Nevertheless, habitat fragmentation and its subsequent loss, followed by a decline in population size that affects mate availability via loss of genetic diversity, will likely negatively impact the long-term persistence of *S. brachyodon*^39^, necessitating active conservation measures especially for population KO. In such cases, population size, genetic diversity, and geographic distance have often been used to make decisions. However, Lawrence and Kaye^40^ suggested that ecological similarity is a better predictor of plant performance and establishment when identifying source populations for reinforcement and translocation actions. Community assemblage analysis of all extant populations showed the greatest similarity between populations OR and KO, while population PE differed the most^31^. Combined with the lower degree of differentiation, caused by moderate gene flow between populations OR and KO found in the present study, population OR appears to be a suitable source population for reinforcement and translocation actions in population KO and neighbouring suitable habitats.

Flower longevity did not differ between populations of *S. brachyodon*, despite considerable differences in site ecology and nectar robber’s abundancy and activity. Similar to *S. gesnerifolia* Lindl. & Paxton^41^, nectar robbers were much more active in drilling holes in young flowers, especially in the population PE, but did not affect flower longevity (Fig. 4). Consistent with the minimum longevity hypothesis^42^ that pollination-induced senescence should be more common in protandrous species^43,44^, unmanipulated flowers (treatment A_s_) of *S. brachyodon* that were protected from insect visitors (Fig. 3a) lasted much longer than open-pollinated (C and PL) or hand-manipulated flowers (A_i_, G and Xe). A similar observation was made by Aximoff and Freitas^45^ in *S. sellowiana* Benth., where bagged and non-manipulated flowers remained functional twice as long as manipulated flowers.

During anthesis, *S. brachyodon* produced nectar during the day and night in a pattern, quantity, and concentration very similar to that of *S. pratensis*^46^. In general, as temperature increases, the rate of nectar secretion also increases^47^. However, in the afternoon, the amount of nectar secreted by flowers of *S. brachyodon* decreased, probably as a result of heat and water stress^48^. This pattern was more pronounced at population PE, where the decrease in nectar secretion was observed as early as midday (Fig. 3b), when plants appeared wilted but recovered during the night.

Significant differences in the composition and abundance of flower visitors between the two sites (Table 2) were not surprising. However, temporal and spatial variations in the quality and quantity of flower visitors (including pollinators) appear to be the rule rather than the exception^49–52^. Despite the fact that results of different studies on the composition and abundance of insect flower visitors in *Salvia* are difficult to compare due to different sampling methods and duration of observation periods^53–58^, it appears that *S. brachyodon* is visited by a large number of insect taxa, of which only *B. argillaceus* effectively triggers the lever mechanism, which, despite the wide variation in flower visitor composition, suggests that *S. brachyodon* is an ecological specialist in which pollination niches are defined by a major pollinator. Consequently, any change in this flower visitor is usually accompanied by a change in the pollination system^59^. Changes in the pollination niches of specialized plants associated with replacement of the main pollinator usually result in adaptive pollinator-mediated divergence in floral traits^60–62^. The fruit set observed in the field and the results of the hand pollination experiment indicate that in *S. brachyodon*, although closely adapted to a single pollinator species, thieves and robbers of nectar and pollen occasionally contribute to pollen transfer, allowing sexual reproduction in the absence of the pollinator.

Of the various life history traits (including the number of fruits and seeds produced), mating systems have the greatest influence on patterns of polymorphism^63^. The high pollen-to-ovule ratio and the results of controlled hand pollination indicate that *S. brachyodon* has a mixed mating system and generally prefers cross-pollination to self-pollination (Fig. 3c). When pollinators were excluded (A_s_), fruit set declined significantly, suggesting that this species requires pollination vectors to ensure reproductive success. Fruit set after autogamous (A_i_) and allogamous treatments (G, Xe, and PL) did not differ significantly in either population, except for the xenogamous treatment (Xe) in population OR, indicating that individuals in this population clearly prefer outcrossing to selfing. Plant mating systems may vary considerably among taxa and populations both spatially and temporally^64–66^ due to a variety of intrinsic and extrinsic factors that often have confounding effects. The low fruit set in control treatment in PE is best explained by a lack of pollinators (Table 2), but can also be a result of nectar robbing (Videos S1 & S2) and herbivory. The effects of nectar robbers are very complex and can affect plant reproductive success in several ways^67,68^. Our results suggest that nectar robbers may have a direct or indirect detrimental or neutral effect on fruit set in *S. brachyodon*. In population PE, four out of nine flower buds (44%) with boreholes drilled by nectar robbers wilted before anthesis, which alone could reduce fruit set by 8% (Fig. 4). In the same population, severe habitat destruction caused by wildfire may have resulted in significant turnover of flower visitors. Here, nectar robbers may have disrupted the pollinator niche that hasn't recovered after the fire, resulting in reduced fruit set, as shown in the ecologically similar *S. fruticosa* Mill.^54,69^. Sparse fruit set in population PE observed in the control treatment and in neighbouring plants not included in the experimental design suggests that thieves and robbers may also contribute directly to pollination success, albeit to a negligible extent.

Flower characteristics differed significantly among populations (Fig. 5a, Table 3), which could be partly explained by differences in site ecology and composition of flower visitors. On the other hand, mating systems have the greatest influence on patterns of polymorphism. Increased isolation between populations results directly from selfing and indirectly from evolutionary changes, i.e., smaller flowers^63^. According to this scenario, selfing populations are more genetically differentiated than outcrossing populations and reduce allocation to attraction^70^. In all controlled pollination treatments, except PL in population OR, seed weight was higher in population OR than in genetically differentiated population PE, although significantly only in treatment Xe in population OR (Fig. 5b). These results are also consistent with the fruit set (Fig. 3c). In general, allogamous treatments in *S. brachyodon* resulted in higher seed weight than autogamous treatments, which was also demonstrated in *S. verbenaca*^71^. On the other hand, seed weight and fruit set in treatments A and G in population PE were not significantly different from treatments PL and C (Figs. 3d and 4), suggesting that most of the pollen flow in population PE occurs between flowers of the same plant. Morphometric analysis of the two populations revealed significantly higher values for vegetative traits (plant height, inflorescence length, number and length of internodes, number and length of primary branches) and lower values for reproductive traits (calyx tube and teeth lenght) for population PE in comparisson to population OR^72^. This is consistent with the results of our morphometric analysis of flowers, where the higher values for flower traits were obtained in population OR, suggesting different strategies of resource allocation between vegetative and reproductive parts in the two populations.

## Conclusions

The results of this multidisciplinary study, which included different types of genetic markers and anthecological analyses, suggest that *Salvia brachyodon* is an ecologically specialised sage exposed to persistent and extensive environmental pressure. Range contraction and habitat fragmentation during the evolutionary history of the species have had a significant impact on present-day genetic structuring, which is associated with strong differences between populations in reproductive biology and survival strategies. Results derived from plastom DNA analysis indicate a recent and severe genetic bottleneck that has erased the majority of the species' genetic diversity. The extant populations have found themselves in habitats that are isolated geographically, reproductively, and genetically. Various ecological factors, in which plant-pollinator interactions play a pivotal role, have likely created selection pressures that have led to genetic and phenotypic differentiation. This recent differentiation, detected by microsatellite markers and coupled with contrasting characteristics in the pollination biology and resource allocation strategies of the species, sheds light on the ongoing and dynamic process of its adaptive divergence.

## Methods

### Study system

Among the European sage species, *Salvia brachyodon* (Fig. 1a) stands out for its large flowers, along with *S. eichlerana* Heldr. ex Halácsy and *S. ringens* Sibth. & Sm. However, it is noteworthy that *S. brachyodon* has relatively shorter calyx teeth compared to the other two species. Morphologically, *S. brachyodon* closely resembles *S. ringens*, an allopatric endemic of the southeastern Balkans. Phylogenetic studies have revealed that both *S. brachyodon* and co-occurring *S. officinalis* L. belong to the *Salvia* subgenus *Salvia*^73,74^. Compared to the evolutionarily closely related and sympatric *S. officinalis*, *S. brachyodon* prefers relatively cooler, moister, deeper, and more nutrient-rich soils that occur on dolomite or dolomitic limestone at higher elevated sites. Their flowering periods also do not overlap. Nowadays, there are only three known populations of *S. brachyodon*, all located in the central part of the coastal Dinaric Alps along the eastern Adriatic Sea (Fig. 1b), all with a small number of individuals: Pelješac Peninsula, Konavle and Mt. Orjen^31^, hereafter referred to as populations PE, KO and OR, respectively. Despite its ability to cope better than *S. officinalis* with interspecific competition through clonal reproduction^75^, populations of *S. brachyodon* are severely threatened by the abandonment of traditional land use and by fire, making the species endangered (EN) according to IUCN criteria^31^. In fact, population KO is on the brink of extinction (personal observation). Although specimens of *S. brachyodon* appear fairly uniform morphologically, Liber et al.^72^ found significant differences in some vegetative and reproductive traits among populations. Individuals in population PE are generally more robust, have a greater number of primary branches and internodes, and longer inflorescences, whereas individuals in population OR have longer calyces. From the point of view of population genetics, population PE was studied in detail by conducting a high-resolution spatial genetic analysis, confirming a high degree of clonality within the population^75^. In 1998, this population suffered a severe wildfire that completely changed the habitat characteristics and community assemblage. Contrary to expectations, a study conducted 15 years after the fire at the same site, showed a slight heterozygote excess, a high level of genetic diversity, as well as clonal reproduction and a genetic bottleneck^76^. Nowadays, *S. brachyodon*, with a peak flowering period in late July and mid-August, is a keystone species and an edificator in four floristically and ecologically well-defined groups of stands distributed in three localities. Although these stands represent different syntaxa, they all belong to the dry eastern (sub)Mediterranean rocky grasslands, which were once more widespread and continuously distributed (Fig. 1c and d). However, their current state varies in terms of successional stages towards (sub)Mediterranean forest vegetation^31^ due to abandonment of traditional landscape management. Population OR occupies stony pastures and grasslands^77^ characterized by shallow calcareous soils derived from Jurassic dolomitic limestone^78,79^. In addition to these habitats, this population can also be observed in fringe vegetation and thermophytic forests containing *Quercus pubescens* Willd., *Carpinus orientalis* Mill., and *Fagus sylvatica* L.^80^. Similarly, population PE is not exclusively confined to stony pastures. It historically thrived on humus-rich soils within stands of Dalmatian Pine (*Pinus nigra* subsp. *dalmatica* (Vis.) Franco), which are situated just above the climazonal evergreen Mediterranean vegetation^81,82^. The geological composition of the bedrock supporting population PE consists of dolomites and limestones dating back to the lower and upper Cretaceous periods^83,84^, while population KO is primarily found on Jurassic dolomitic limestone (ibid.). The mean annual temperature in *S. brachyodon* sites varies, ranging from 12–13 °C in populations PE and KO to 10–11 °C in population OR^85^. The amount of annual precipitation increases in a west–east direction, with populations PE, KO, and OR receiving approximately 1300–1400 mm, 1500–1750 mm^86^, and more than 3700 mm^87^, respectively. The precipitation regime in these areas follows a Mediterranean pattern.

### Population genetics

In total, leaf material from 265 individuals was collected in 2015: 119, 70 and 76 from populations PE, KO and OR, respectively. After rapid desiccation of the plant material in silica gel, DNA was extracted using GenElute plant genomic DNA miniprep kit (Sigma-Aldrich, St. Louis, MO, USA). The set of analysed loci, the PCR procedure, and genotyping, were as described in Radosavljević et al.^75^. To identify multi-locus genotypes (MLG) and to assess the levels of clonality (genotypic richness (*R*)), RClone package version 1.0.2^88^ was used. After the identification of individuals (i.e., ramets) that shared the same genotypes and before the further analysis of populations’ structure and diversity, replicates were removed from the dataset. After the identification of the MLGs, to assess the phylogeographic structure of the species, two regions of the plastome genome were analysed, *rps*16*-trn*K and *rpl*32*-trn*L. These regions were selected based on recently published results^32^, where they were characterized by significant variability in the closely related *S. officinalis*. For this purpose, 20 MLGs from populations PE and KO, and 10 MLGs from population OR were randomly selected. PCR reactions were performed in a total volume of 15 μL containing 8 μL Nuclease-Free Water (Qiagen®), 1.5 μL 10 × PCR buffer (TaKaRa Taq™ Hot Start Version), 1.2 μL dNTP solution (2.5 mM each, TaKaRa TaqTM Hot Start Version), 0.6 μL 5 μM of each forward and reverse primer, 0.1 μL TaqHS polymerase (5 U/μL, TaKaRa TaqTM Hot Start Version) and 3 μL of 1 ng/μL DNA. The PCR conditions were as follows: 94 °C for 5 min, followed by 30 cycles of 95 °C for 1 min, 50 °C for 1 min and 65 °C for 4 min, and a final elongation step of 5 min at 65 °C. Exonuclease I (Thermo Scientific™) and FastAP Thermosensitive Alkaline Phosphatase (Thermo Scientific™) was used for the purification of PCR products. The sequencing of the amplicons in both directions was performed using an ABI 3730XL analyser (Applied Biosystems). Sequences were cleaned with Codon Code Aligner v. 9.0.1 (CodonCode Corporation, Dedham, USA) and aligned using the sequence alignment program ClustalW implemented in the MEGAX software^89,90^. Sequences were deposited in GenBank.

### Flower life span and sexual functioning

Flower longevity was investigated by marking a total of 97 flowers, with 45 and 52 from populations PE and OR, respectively, on multiple individuals prior to their opening. Subsequently, the newly opened flowers were observed daily at 8^00^, 14^00^, and 19^00^ h until senescence, and the duration of each flower was recorded in hours. The presence and first appearance of the hole in the corolla tube just above the calyx teeth caused by nectar robbers, a damage that could affect flower longevity, was recorded for different stages of flower anthesis. We assessed pollen viability in upper and lower thecae throughout the flower lifespan using diaminobenzidine reaction (DAB)^91^, assuming that viable pollen, unlike dead and aborted pollen, turns dark reddish brown due to enzyme activity^92^. The effectiveness of the staining was first tested on pollen killed by high temperature. When evaluating the percentage of pollen stained, at least 200 grains (and up to 400) per flower were counted. Since the stigma viability in *Salvia* generally correlates with the opening of the stigma lobes^93^, we first calibrated the intensity of staining of the stigma receptive surface using DAB test and stigma bifurcation using five grade scale for staining (1—stigma not stained, 2—stained only the tips of stigma, 3—at least half of the stigma stained, 4—more than half of the stigma stained, 5—stigma fully stained) and a three-point scale (1—stigma closed, 2—stigma lobes half opened, 3—stigma lobes fully opened) for stigma opening. Stigma receptivity was then assessed by using results of both staining and opening of lobes. A total of 82 and 22 flowers were used to evaluate pollen viability and stigma receptivity, respectively. Both pollen viability and stigma receptivity were tested in population OR.

### Floral rewards

Based on our initial field observations, it became evident that flower visitors of *S. brachyodon* engage in foraging for nectar and/or pollen. Consequently, we proceeded to quantify both aspects. To assess daily nectar production, we selected a random sample of flower buds (69 from population PE and 37 from population OR) belonging to at least 20 different genets per population. This selection aimed to minimize potential damage caused by insect visitors. The chosen flower buds were individually enclosed in tulle bags 24 h before their expected opening, ensuring the exclusion of nectar collection by insect visitors. Subsequently, the bagged flowers were carefully opened, and nectar was extracted using 5 µl microcapillaries (Drummond) at three different time intervals: 6^00^–7^00^ h, 12^00^–14^00^ h, and 17^00^–19^00^ h. The length of the nectar column was measured using digital callipers. Once the nectar collection was completed, the bagged flowers were resealed to prevent further nectar removal or contamination. To determine nectar concentration, the collected nectar was dispensed onto a handheld portable refractometer (0–50°Brix, Eclipse Professional, Bellingham and Stanley Ltd), and the % Brix (i.e. g sucrose per 100 g solution) was recorded. The nectar standing crop was determined by measuring a total of 31 well-preserved and freshly opened flowers (from the population PE) at 8^00^ h and 19 flowers (from the population OR) at 14^00^ h. These flowers were randomly selected from several genets per population to ensure representativeness.

### Mating system, pollen limitation and pollen-to-ovule ratio

Pollination ecology and floral biology studies on *Salvia brachyodon* were performed in situ only in populations PE and OR due to a low number of individuals in population KO. Buds selected for treatments were covered with a tulle to prevent insect interactions. The following treatments were applied to determine the mating system, effects of insect exclusion and pollen source on fruit production: (a) spontaneous autogamy (A_s_)—individual flowers covered with a tulle were left intact; (b) induced autogamy (A_i_)—individual flowers were pollinated with their own pollen, then emasculated by cutting of the anthers and covered again with the tulle; (c) geitonogamy (G)—individual flowers were carefully emasculated in the bud, pollinated with pollen from flowers of the same plant and then covered again with a tulle; (d) xenogamy (Xe)—individual flowers were emasculated in the bud, pollinated with a fresh pollen mixture collected from several different plants from the same population and covered again with a tulle; (e) supplementary pollination (PL)—flowers were pollinated with a fresh pollen mixture collected from several individual plants and left uncovered; (f) control (C)—flowers were labelled and left intact. A total of 288 (PE) and 175 (OR) flowers were included in the treatments. After 30 days, fruit production was recorded. From the results of hand pollinations, we calculated index of inbreeding depression (*δ*_*i*_) related to the mating system. To distinguish possible effects of dichogamy from genetic incompatibility, autogamous performance was determined from the results of geitonogamous pollen transfer. Hence, the degree of inbreeding depression was determined by the ratio between fruit set and reproductive success of geitonogamously (*wG*) and xenogamously pollinated flowers (*wXe*), as suggested by Charlesworth and Charlesworth^94^: *δ*_*i*_ = *1* − *(wG/wXe)*.

To estimate the pollen-to-ovule ratio (P/O ratio) and overall pollen production, we counted pollen using a Fuchs-Rosenthal counting chamber; we took 10 µl of the pollen suspension from a single thecae and filled the chamber with a volume of 0.2 µl. Pollen grains were counted and then the number of pollen grains per anther and flower was calculated. We performed 10 replicates with one upper and one lower thecae per flower. P/O ratio as the total number of pollen grains produced per flower divided by the number of ovules was then calculated as a proxy for the breeding system^95^. The specific number of manipulations in studies on sexual functioning, floral rewards and mating system is indicated in Figs. 3 and 5.

### Flower visitors

The assemblage and behaviour of flower visitors were assessed by direct observations, video recordings, and net catches during the whole time of investigation from July 20 to July 27 2016 in population PE and from July13 to July 19 2016 in population OR. If necessary, visitors were captured for identification and subsequently preserved in ethyl acetate for later dissection and identification in the laboratory. Voucher specimens are deposited at the Natural History Museum Rijeka. Observation periods were determined based on the previous years’ experience of insect behaviour, visitation frequency, and dynamics during the day. Quantitative observations, involving the count of visitors, as well as qualitative observations, documenting the specific taxa visited and their corresponding behaviors, were conducted within 4 m^2^ quadrats with homogenous plant cover. These observations took place on sunny and windless days during the main flowering period, specifically between 7^00^–9^00^, 10^00^–12^00^, 13^00^–15^00^ and 16^00^–18^00^ h GMT. The observations were carried out on July 13–14, 2017, in the OR population and on July 20–21, 2017, in the PE population, resulting in a total of 32 h of observation. Flower visitors were classified as legitimate visitors if they entered the corolla through the corolla tube, or illegitimate if they accessed pollen or nectar by other means. Visitors were further classified as pollen (PT) or nectar thieves (NT) if they legitimately entered the flower to search for pollen or nectar without touching the anthers and/or stigma, and as nectar robbers (NR) if they gained access to nectar through a hole in the corolla tube^96^. Legitimate visitors who effectively triggered the staminal lever mechanism and contacted both the thecae and the stigma surface were classified as pollinators (P).

### Flower morphology and seed weight

To examine potential resource allocation among reproductive parts between populations, we measured 16 quantitative floral traits in situ using a digital calliper (see Table 3) to estimate flower size and functioning depending on morphology. The selection of specific traits for measurement can have a significant impact on pollinator composition and efficiency in *Salvia* spp. ^56,58^. Seeds obtained from controlled hand pollination in populations PE and OR were weighed to the nearest 10 μg (Ohaus AP250D) in order to check whether seed weight depend on pollen source (selfing vs. outcrossing) and for the differences between populations. Voucher specimens are deposited in the herbarium of the Natural History Museum Rijeka (NHMR).

### Statistical analyses

Population genetic datasets were analysed using different statistical tools. To estimate observed and expected heterozygosity and inbreeding coefficient, we used GENEPOP software^97^. R packages “PopGenReport”^98^ and “hierfstat”^99^ were used to assess the allelic richness and pairwise *F*_st_ values, respectively. For the assessment of the genetic structure, in order to elucidate the structuring patterns and to make certain assumptions about the data, the principal component analysis (PCA) was performed as implemented in the R package “adegenet”^100^, followed by the Bayesian assignment analysis using STRUCTURE software ver. 2.3.3^101^. Thirty runs per cluster (K), with K ranging from 1 to 4, were performed. Each run consisted of a burn-in period of 200 000 steps followed by 1 000 000 MCMC replicates. To process the obtained results and to select the most likely number of clusters following Evanno et al.^102^, STRUCTURE HARVESTER v0.6.92.^103^ was used. Finally, runs were clustered and averaged using CLUMPAK^104^. Gene flow between populations (*N*_m_) was calculated from the pairwise *F*_st_ values using the formula: *N*_m_ = (1 − *F*_st_)/4 *F*_st_^105^.

The data on floral biology and mating system were subjected to analysis using generalized linear models (GLM) with fruit set as the response variable and treatments and populations as predictors, considering all populations and each population separately. The response variable was fitted to a binomial distribution with a logit link function. Likelihood ratio test was performed to compare the full model with a restricted model and by calculating p values using the χ^2^ distribution. Differences between levels of each effect were analysed post hoc by multiple comparisons of means with Tukey contrasts, adjusting data for normality and testing for homogeneity of variance. All statistical analyses were performed using the R version 3.1.1^106^ and packages ‘stats’, ‘car’, and ‘multcomp.

Differences in stigmatic receptivity and pollen germinability along the flower lifespan were investigated using GLM with the response variables (staining/no staining for stigma receptivity and pollen germinability) being adjusted to a binomial distribution and a logit link function.

Nectar volume and sugar concentration were adjusted to a Gaussian distribution with an identity link function.

Data on mating system were analysed with fruit set as the response variable and manipulations as predictors, considering all populations and each population separately. The response variable was fitted to a binomial distribution with a logit link function.

We examined the dominance, evenness, and diversity of pollinators across populations, employing a range of indices. To compare these indices, we employed a permutation test for equality. Additionally, we assessed differences in flower visitor fauna using the χ2 test implemented in PAST^107^. Results were considered significant if the probability for the null hypothesis was below 0.05.

To analyse the differences in flower and seed characteristics, we performed the Kruskal–Wallis test, as the data sets did not meet the assumptions of normality and homogeneity of variance. This non-parametric test allowed us to assess the equality of medians. Subsequent post-hoc pairwise comparisons were conducted through the Mann–Whitney test using PAST. Statistical significance was determined if the probability for the null hypothesis fell below 0.05, with *p*-values adjusted using the Bonferroni correction for multiple comparisons.

### Ethical approval

Formal identification of the plant material (*Salvia brachyodon*) was provided by Boštjan Surina. Voucher specimens of the plant material used in the study have been deposited in a publically available herbarium at the Natural History Museum Rijeka (Index Herbariorum acronym NHMR: https://sweetgum.nybg.org/science/ih/herbarium-details/?irn=157889). Deposition (voucher) numbers are included in the manuscript (Table 1). Permission to perform the research and to collect voucher specimens of *Salvia brachyodon* was issued by Nature Protection Directorate of the Ministry of Economy and Sustainable Development of the Republic of Croatia (UP/I-352-04/22-08/15, 517-10-1-1-22-4). All the experimental research and field studies on study plant complied with relevant institutional, national, and international guidelines and legislation.

### Supplementary Information

Supplementary Table 1.

Supplementary Table 2.

Supplementary Video 1.

Supplementary Video 2.

Supplementary Video 3.

Supplementary Video 4.

Supplementary Legends.

## Data Availability

Sequences generated in this study are available in NCBI GenBank under accession numbers OQ067501, OQ067504, OQ067506 (*rps*16*-trn*K region) and OQ067510, OQ067513, OQ067515 (*rpl*32*-trn*L region). Other datasets supporting the conclusions of this article are available in the Zenodo repository, 10.5281/zenodo.7457749.
