# Supplementary material for: Lack of pollinators selects for increased selfing, restricted gene flow and resource allocation in the rare Mediterranean sage Salvia brachyodon
Source: Sci Rep. 2024 Feb 29;14:5017. doi: 10.1038/s41598-024-55344-7 (PMC10904396; doi:10.1038/s41598-024-55344-7)
Supplement: Supplementary file 1 — Supplementary Table 1. [file 41598_2024_55344_MOESM1_ESM.docx]

**Additional file 1: Table S1.** Generalized linear modelling of controlled hand pollination treatments with *Salvia brachyodon* for populations Pelješac peninsula (PE) and Mt. Orjen (OR) –multiple comparisons of means (Tukey Contrasts).

|  | populations | PE | | | | | | | | | OR | | | | | | | | | | |
| --- | --- | --- | --- | --- | --- | --- | --- | --- | --- | --- | --- | --- | --- | --- | --- | --- | --- | --- | --- | --- | --- |
|  | treatments | A_s_ | G | Xe | | PL | | C | | A_s_ | | A_i_ | G | | Xe | | PL | | C | |  |
| PE | A_s_ |  | * | | * | | ** | | 1 | | 1 | * | | ** | | *** | | *** | | *** | |
|  | G | 3.48 |  | | 1 | | 0.97 | | *** | | * | 1 | | 1 | | * | | 0.81 | | 0.10 | |
|  | Xe | 3.53 | -0.34 | |  | | 0.79 | | *** | | * | 1 | | 1 | | ** | | 0.47 | | * | |
|  | PL | 4.19 | 1.29 | | -1.74 | |  | | *** | | ** | 0.86 | | 1 | | 0.61 | | 1 | | 0.74 | |
|  | C | -0.78 | 4.95 | | 5.19 | | 5.58 | |  | | 1 | ******* | | *** | | *** | | *** | | *** | |
| OR | A_s_ | -0.31 | 3.34 | | 3.34 | | 4.25 | | -0.37 | |  | * | | * | | *** | | *** | | *** | |
|  | A_i_ | -3.07 | 0.45 | | 0.21 | | 1.63 | | -4.43 | | -3.00 |  | | 1 | | * | | 0.60 | | ***** | |
|  | G | 3.70 | 0.50 | | -0.88 | | 0.77 | | 5.10 | | 3.55 | 0.89 | |  | | 0.16 | | 0.98 | | 0.29 | |
|  | Xe | 5.51 | 3.28 | | 3.81 | | 2.02 | | 6.83 | | 5.22 | 3.51 | | 2.76 | |  | | 0.84 | | 1 | |
|  | PL | 4.48 | 1.71 | | -2.22 | | 0.40 | | 5.89 | | 4.25 | 2.03 | | 1.17 | | 1.67 | |  | | 0.91 | |
|  | C | 5.14 | -2.94 | | -3.35 | | -1.84 | | 6.30 | | 4.94 | 3.17 | | -2.49 | | 0.00 | | -1.52 | |  | |

Lower left handed corner–z-values, upper right handed corner–p-values. A_s_–spontaneous selfing, A_i_–induced selfing, G–geitonogamy, Xe–xenogamy, PL–pollen limitation, C–control. Red color indicates near statistical significance; (χ^2^ = 180.47, df. = 10, p < 0.0001); *p < 0.05, **p < 0.01, ***p < 0.001
