# Supplementary material for: Lack of pollinators selects for increased selfing, restricted gene flow and resource allocation in the rare Mediterranean sage Salvia brachyodon
Source: Sci Rep. 2024 Feb 29;14:5017. doi: 10.1038/s41598-024-55344-7 (PMC10904396; doi:10.1038/s41598-024-55344-7)
Supplement: Supplementary file 2 — Supplementary Table 2. [file 41598_2024_55344_MOESM2_ESM.docx]

**Additional file 2: Table S2**. Seed weight (mg) of *Salvia brachydon* according to various pollination treatments in populations on Pelješac peninsula (PE) and Mt. Orjen (OR).

| Populations | Pelješac peninsula (PE) | | | | |  | Mt. Orjen (OR) | | | | |
| --- | --- | --- | --- | --- | --- | --- | --- | --- | --- | --- | --- |
| treatments | A | G | Xe | PL | C |  | A | G | Xe | PL | C |
| Number of seeds measured | 7 | 31 | 77 | 32 | 9 |  | 22 | 28 | 46 | 26 | 30 |
| Min | 1,1 | 0,5 | 0,8 | 0,1 | 1,5 |  | 0,6 | 0,1 | 2,3 | 0,1 | 0,6 |
| Max | 6,7 | 9,2 | 9,7 | 9,5 | 5 |  | 12,4 | 11,8 | 11,6 | 9,1 | 11,8 |
| Mean | 2,9 | 3,4 | 4,9 | 4,1 | 3,2 |  | 4,8 | 3,7 | 7,4 | 2,7 | 4,7 |
| Std. error | 0,7 | 0,4 | 0,2 | 0,4 | 0,4 |  | 0,7 | 0,6 | 0,4 | 0,5 | 0,7 |
| Stand. dev | 1,8 | 2,1 | 2,0 | 2,4 | 1,3 |  | 3,2 | 3,2 | 3,0 | 2,4 | 3,6 |
| Median | 2,4 | 2,6 | 5,0 | 3,7 | 3,5 |  | 4,3 | 2,8 | 8,7 | 2,0 | 2,9 |
| Coeff. var. (%) | 61,6 | 63,2 | 40,2 | 57,8 | 41,7 |  | 66,7 | 86,7 | 41,0 | 89,2 | 77,4 |

Treatments: A–autogamy, G–geitonogamy, Xe–xenogamy, PL–pollen limitation, C–control.
