# Supplementary material for: Lack of pollinators selects for increased selfing, restricted gene flow and resource allocation in the rare Mediterranean sage Salvia brachyodon
Source: Sci Rep. 2024 Feb 29;14:5017. doi: 10.1038/s41598-024-55344-7 (PMC10904396; doi:10.1038/s41598-024-55344-7)
Supplement: Supplementary file 7 — Supplementary Legends. [file 41598_2024_55344_MOESM7_ESM.docx]

**Supplementary file captions**

**Additional file 1: Table S1**. Generalized linear modelling of controlled hand pollination treatments.

**Additional file 2: Video S1**. Nectar robbing by an individual of *Xylocopa violacea.*

**Additional file 3: Video S2**. Nectar robbing by an individual of *Bombus terrestris.*

**Additional file 4: Video S3**. Effective triggering of staminal lever mechanism by an individual of *Bombus argillaceus.*

**Additional file 5: Video S4**. Pollen robbing by an individual of *Episyrphus balteatus*.

**Additional file 6: Table S2**. Seed weight (mg) of *Salvia brachydon* according to various pollination treatments.
